# Evaluation of Nipple–Areola Circulation in Central Pedicle Mastopexy Patients with Subpectoral Implant Placement: A Quasi-Experimental Study

**DOI:** 10.1007/s00266-024-04113-y

**Published:** 2024-06-06

**Authors:** Fatma Nilay Tutak, Ozan Balık, Semra Bulbuloglu

**Affiliations:** 1 Department of Research and Development, Vetal Animal Health Products Joint Stock Company, Adiyaman, Turkey; 2 Clinic of Plastic Reconstructive and Aesthetic Surgery by Ozan Balık, Levent, Besiktas, Istanbul, Turkey; 3 Division of Surgical Nursing, Department of Nursing, Faculty of Health Sciences, Istanbul Aydin University, Istanbul, Turkey

**Keywords:** Central pedicle, Mastopexy, Nipple–areola circulation, Subpectoral reconstruction

## Abstract

**Objective:**

Mastopexy is a procedure which is used in breast lift and reconstruction surgery and requires a small amount of parenchymal resection. In this procedure, the preservation of nipple–areola circulation is vital. The purpose of this study is to evaluate nipple–areola circulation in patients undergoing central pedicle mastopexy with subpectoral implant placement.

**Material and Method:**

In this observational quasi-experimental study, data were collected retrospectively from electronic medical records. The perioperative nipple–areola circulation of patients undergoing central pedicle mastopexy with subpectoral implant placement was evaluated by integrated laser Doppler flowmetry. Descriptive statistics, one-way analysis of variance, and Tukey’s range tests were used to analyze the data.

**Results:**

The preoperative, skin dissection, pectoral elevation, implant placement, 24th hour, and 2nd week nipple–areola circulation statuses of each patient who underwent central pedicle mastopexy with subpectoral implant placement were examined using an integrated laser Doppler flowmeter, and the results were compared. At each stage, all measurements were in the range of 1.8–3.6 ml/min/100g. There was no statistically significant difference between the measurement results.

**Conclusion:**

Central pedicle mastopexy with subpectoral implant placement seems highly advantageous in terms of better functionality and aesthetics in the reconstruction of heavy and sagging breasts.

**No Level Assigned:**

This journal requires that authors assign a level of evidence to each submission to which Evidence-Based Medicine rankings are applicable. This excludes Review Articles, Book Reviews, and manuscripts that concern Basic Science, Animal Studies, Cadaver Studies, and Experimental Studies. For a full description of these Evidence-Based Medicine ratings, please refer to the Table of Contents or the online Instructions to Authors www.springer.com/0026

**Supplementary Information:**

The online version contains supplementary material available at 10.1007/s00266-024-04113-y.

## Introduction

Although mastopexy is a surgical intervention that is similar to reduction mammoplasty, it is associated with a smaller parenchymal resection [1]. It is thought that women who are obese (body mass index (BMI) > 30) or morbidly obese (BMI > 40) can have smaller breasts by losing weight. Because obese individuals have a higher risk of surgical complications [2], it is argued that lowering BMI below 30 would be a healthier approach for breast reduction [3, 4]. Nevertheless, the increased physical load caused by breast hypertrophy usually justifies the need for reduction mammoplasty [5, 6]. Additionally, mastopexy operations are carried out for reconstruction purposes to resolve issues such as burns, trauma, or the sagging of the breast tissue over time [7]. Mastopexy and reduction mammoplasty have common techniques in terms of skin excision patterns and pedicle formation. However, higher amounts of breast tissue can be resected in reduction mammoplasty [8].

In mastopexy, vertical and horizontal bipedicle techniques are used in addition to inferior, superior, lateral, medial, and central mound pedicles. In the central mound pedicle mastopexy technique, the thick skin around the nipple and the subcutaneous flap are dissected, and surrounding tissues are reduced to the desired extent by taking the nipple in the middle as a central reference [1, 8]. The vascular structure of the breasts includes the thoracodorsal artery, intercostal perforators, internal mammary artery, thoracoacromial artery, and lateral thoracic artery [9]. The two most important sources of vascularization are the internal mammary artery and the lateral thoracic artery [10, 11]. The nipple–areola region is innervated by the anterior and lateral cutaneous branches of the third and fifth intercostal nerves, mostly by the lateral cutaneous branch of the fourth intercostal nerve [12]. The safest pedicle for nipple–areola circulation in mastopexy operations is debated [13–15].

In central pedicle mastopexy with subpectoral implant placement, the success of the pedicle is indicated by its expansion to provide good activation of vascularization and prevent sensory loss in the nipple–areola. In this study, it was aimed to evaluate nipple–areola circulation in patients undergoing central pedicle mastopexy with subpectoral implant placement.

## Material and Method

This study was carried out with an observational and quasi-experimental design to evaluate the nipple–areola circulation statuses of patients undergoing central pedicle mastopexy with subpectoral implant placement.

### Design and Participants

After obtaining ethics committee approval, this study was carried out with the participation of patients who underwent central pedicle mastopexy with subpectoral implant placement at a Plastic, Reconstructive, and Aesthetic Surgery Clinic in Western Turkey. The sample consisted of *n *= 11 women who met the inclusion criteria.

### Inclusion and Exclusion Criteria

The inclusion criteria for the study were (1) undergoing central pedicle mastopexy with subpectoral implant placement at the clinic where the study was conducted, (2) being a woman between the ages of 18 and 55, (3) having no disease/comorbidity that would lead to any circulation disorder, and (4) not using any medication related to conditions of the cardiovascular system. The sample of the study excluded patients who had the opposite characteristics of any of the inclusion criteria and/or those who smoked and/or consumed alcohol, as well as mastopexy patients who underwent glandular excision.

### Data Collection Tools and Procedure

At the clinic where the study was carried out, in the routine health examinations of patients, the circulation status of the nipple–areola is evaluated before and after mastopexy operations using the integrated laser Doppler flowmetry method, and the outcomes are recorded. In this study, data were collected retrospectively from the electronic medical records of the patients. Results of measurements made using an integrated laser Doppler flowmeter extracted from the electronic medical records were noted on the data recording form prepared by the researchers. The ages and health-related data of the patients (smoking and alcohol consumption status, comorbid and chronic diseases, medications used) were also recorded.

### Incision Site and Surgical Technique

**Fig. 1:**
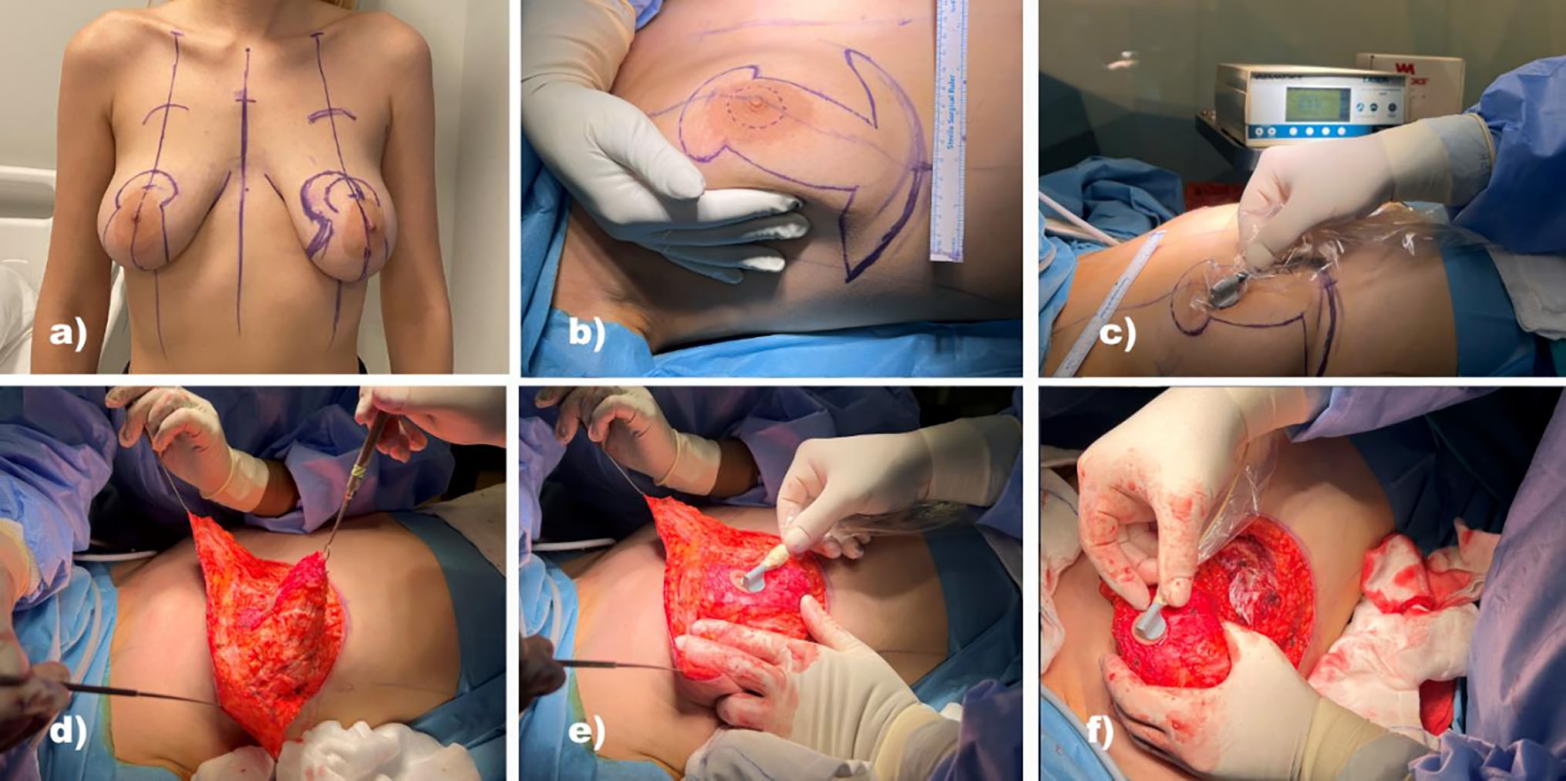
Appearance of the breast before and after mastopexy. A pouch was opened with an incision made from the inferior of the pedicle without crossing the pectoral muscle. Pectoral muscle elevation was facilitated on the Würinger septum projection. Dissection continued up to 2 cm to the boundary of the sternum in the medial and until serratus anterior fibers were left behind in the lateral

For all patients, augmentation was planned with inverted T scars, central pedicle mastopexy, and round silicone implants. During mastopexy, an incision was made using a technique in accordance with Wise patterns, and the surgery was completed with an inverted T scar. The implants that were used had volumes varying in the range of 275–400 cc. Skin flaps were left at approximately 1 cm, and dissection progressed up to a 1 cm distance from the pectoral muscle fascia around the central pedicle. The pectoral muscle was not crossed in any of the patients. An incision was made at the inferior border of the pectoral muscle, the submuscular region was reached through the incision, a pouch was opened, and the implant was placed in this pouch. Pectoral muscle elevation was achieved by accessing the pectoral muscle on the Würinger septum projection. While dissection continued until it surpassed the serratus anterior fibers in the lateral, it continued up to a 2 cm distance from the edge of the sternum in the medial (Figure 1).

### Integrated Laser Doppler Flowmetry

In this study, to identify the presence of hyperemic response before and after mastopexy, we examined the cutaneous blood circulation of the nipple and areola. The measurements were made in a quiet room with an ambient temperature of 21–23 °C. Each patient was allowed to rest in the supine position for 20 min. The breasts and infraclavicular regions were exposed, and antisepsis was facilitated using a 0.9% isotonic sodium chloride solution. The integrated laser Doppler flowmeter (Laser Flo BPM2, Vasamedic, St Paul, MN, USA) and an iontophoretic drug delivery probe containing a temperature regulator were placed on the region below the infraclavicular area over the 3rd or 4th intercostal space along the medical clavicular line (with a conductive hydrogel dispersive electrode at approximately 15 cm). The laser Doppler flowmeter continuously measured cutaneous blood flow and red blood cell flux. The Brownian motility standard solution was used to calibrate the device. The temperature placed around the probe kept the local skin temperature at 33°C throughout the testing procedure. The rate, duration, and intervals of blood flow were recorded. A USB power supply (PF 751, Perimed, Järfälla, Sweden) connected to the drug dispersive probe and the drug delivery probe was used for charging. During the measurements, the arm was kept at the heart level, and blood pressure was measured by photoplethysmography (Finometer PRO, Finapres Medical Systems, Amsterdam, Netherlands).

### Statistical Analysis

The Statistical Package for the Social Sciences (SPSS) 25.0 IBM (Armonk, NY) program was used to analyze the data. Regardless of normality test results, descriptive statistics (mean, standard deviation, minimum, and maximum values) were calculated, and one-way analysis of variance (ANOVA) tests were conducted. Tukey’s range test was used to identify the source of significant differences among mean values. The results were interpreted in a 95% confidence interval and at a significance level of *p *< 0.05.

### Ethical Aspects

Before collecting data, the researchers obtained Institutional Review Board approval from a private Plastic, Reconstructive, and Aesthetic Surgery Clinic in Istanbul. After this, each patient provided verbal and written informed consent.

## Results

Table 1 presents the results of the comparisons of the integrated laser Doppler flowmetry measurements of the patients. The mean preoperative, skin dissection, pectoral elevation, implant placement, 24th hour, and 2nd week measurements were, respectively, 2.75 ± 0.45 ml/min/100g, 2.45 ± 0.55 ml/min/100g, 2.6 ± 0.48 ml/min/100g, 2.61 ± 0.39 ml/min/100g, 2.49 ± 0.41 ml/min/100g, and 2.47 ± 0.45 ml/min/100g, and there was no significant difference among the measurements made at different time points (*p *> 0.05).

**Table 1:** Integrated laser Doppler flowmetry measurement results

Measurement time	*X* ± SD	Min, max	Intergroup comparison	
			Test and sig.	Difference test (*r*)
Preoperative	2.75 ± 0.45	2.08, 3.45	*F* = 1.004 *p *= 0.547	–
Skin dissection	2.45 ± 0.55	1.8, 3.6		
Pectoral elevation	2.6 ± 0.48	2, 3.4		
Implant placement	2.61 ± 0.39	2.4, 3.4		
24th Hour	2.49 ± 0.41	2, 3.6		
2nd Week	2.47 ± 0.45	1.8, 2.7		

*p *< 0.05,* F* = One-way ANOVA,* r* = Tukey’s range test

**Fig. 2:**
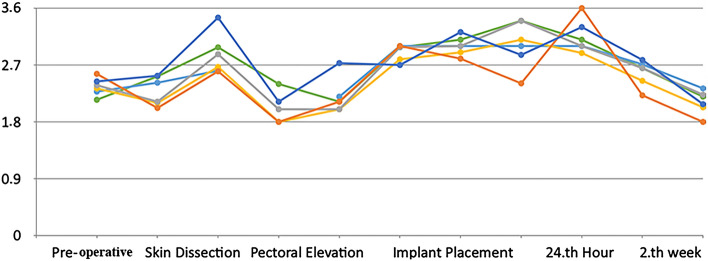
Integrated laser Doppler flowmetry measurements at different time points. The nipple–areola circulation status of each patient undergoing central pedicle mastopexy with subpectoral implant placement was evaluated preoperatively, during skin dissection, during pectoral elevation, during implant placement, at the postoperative 24th hour, and at the postoperative 2nd week by integrated laser Doppler flowmetry

Figure 2 shows the integrated laser Doppler flowmetry measurements of the patients made at the aforementioned time points. It is seen that all measurement results were in the range of 1.8–3.6 ml/min/100g.

**Fig. 3:**
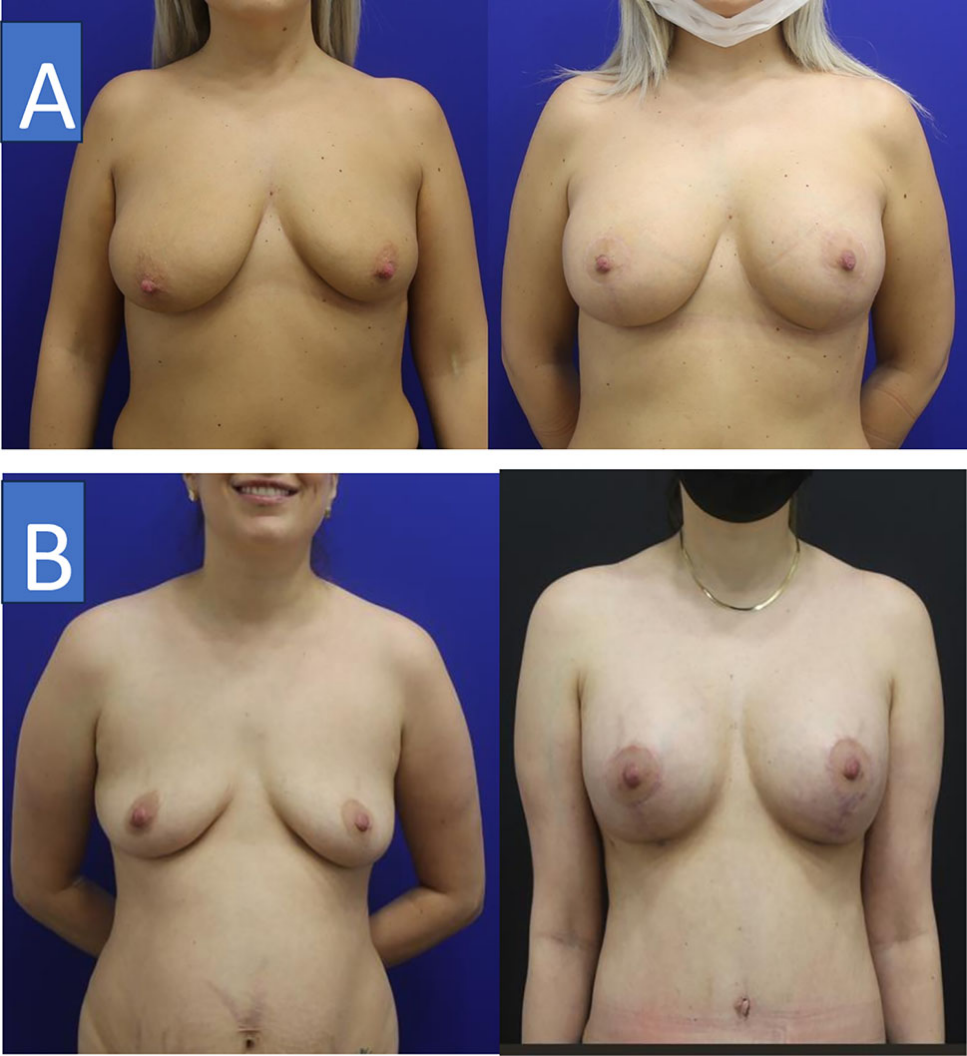
Aesthetic and functional outcomes of central pedicle mastopexy with subpectoral implant placement. Patient A: The 32-year-old patient who had sagging breasts with her nipple–areola 6 cm below normal (left) underwent bilateral central pedicle mastopexy with 400 cc subpectoral implant placement. The breasts were lifted, and the nipple–areola was brought up by 6 cm (right). Patient B: The 38-year-old patient who had sagging breasts with her nipple–areola 3 cm below normal (left) underwent bilateral central pedicle mastopexy with 325 cc subpectoral implant placement. The breasts were lifted, and the nipple–areola was brought up by 3 cm (right)

Figure 3 displays the aesthetic and functional outcomes of the central pedicle mastopexy operations with subpectoral implant placement. It is seen that the breasts had a more aesthetic and forward-facing appearance, the nipple–areola was in its normal position, and there was minimal scar visibility.

## Discussion

Various mastopexy procedures are performed by plastic surgeons to create a more aesthetically pleasing appearance of the breasts and improve their functional status. These procedures can be listed as vertical, buttonhole, primary doughnut, and Wise pattern mastopexy operations [16–18]. Mastopexy procedures are surgeries that usually include reconstruction following nipple-sparing mastectomy and are performed to reduce/augment/fill and/or lift the breast for a broad spectrum of indications [19–21]. In all such procedures, it is crucial to ensure that the nipple–areola of each breast is correctly positioned without drooping. In this sense, central pedicle mastopexy with subpectoral implant placement appears to be highly advantageous.

In comparison with suprapectoral reconstruction, central pedicle mastopexy with subpectoral implant placement involves the lifting of the nipple–areola. Moreover, due to the tension of the infraclavicular part of the pectoralis major muscle, the displacement of a subpectoral implant is very unlikely. Furthermore, an excessively elevated positioning of the nipple–areola may also require a second reconstruction [22]. In cases of poorly positioned nipple–areola, insufficient collateral capsular circulation can disrupt the vascularization of the nipple–areola. This is why subpectoral implant placement is frequently preferred. In this study, a decrease in circulation was observed following the elevation of the pectoral muscle for implant placement, but the amount of this decrease was not statistically significant (*p *< 0.05). In central pedicle mastopexy operations, a vascular area based in the breast provides safe surgical outcomes. In this study, nipple–areola circulation was measured to be lower following skin dissection. This result supported the results of similar studies in the literature [23].

Additional mastopexy operations can be needed in some patients with asymmetric breast structures, overly large breasts, or ptotic breasts. To prevent the development of skin complications, the incision is closed from under the medial part of the infraclavicular area. If a large implant is used or if there is a dimensional difference between the implant size and the surface area of the breast in which it is going to be placed, excessive tension can result in the necrosis of the skin, and this can lead to loss of skin tissue [24–26]. This issue should be kept in mind when selecting implants for patients scheduled for reduction mammoplasty or mastopexy operations.

During central pedicle mastopexy procedures performed simultaneously with subpectoral implant placement, the circulation in skin flaps and the nipple–areola can decrease due to the broad dissection of the skin and the mechanical expansion effect of the implant. On the other hand, according to the concept of “angiosome” proposed by Taylor and Palmer, it is known that tissue perfusion would be reinforced by the presence of “choke” vessels and the activation of these vessels secondary to hypoxia in the further phases [27, 28]. In this study, the lowest nipple–areola circulation measurements were found at the 2nd week following the procedure and following skin dissection in the intraoperative stage, but these values were not significantly lower than the measurement values obtained at other time points (*p *> 0.05).

The indication for central pedicle mastopexy with subpectoral implant placement is the aim to provide an aesthetically pleasing appearance and functionality to the breast. Women who are satisfied with the size of their breasts but avoid mastopexy to prevent scar formation, while still having sagging breasts, may have complaints about posture disorders, the inability to keep their shoulders straight, concerns about going outside without a bra, and feelings of pain and heaviness. In women with these characteristics, reducing the breasts and placing implants suitable for the surface areas of the breasts inside the subpectoral region will improve quality of life substantially and increase self-esteem. The limitations of our study included the fact that it was a single-center study, its sample size was limited, and it did not evaluate long-term outcomes. The presence of moderate ptosis in the cases included in this study may be considered another limitation.

## Conclusion

The advantages of central pedicle mastopexy with subpectoral implant placement are being understood rather recently. Nonetheless, when breast lifting and reconstruction are needed, central pedicle mastopexy with subpectoral implant placement is a highly effective and safe option for providing breasts with an aesthetically pleasing appearance and eliminating dysfunctions caused by overly large and/or sagging breasts.

## Supplementary Information

Below is the link to the electronic supplementary material.

Supplementary file1 (XML 0 KB)

## Declarations

### Data Availability

The datasets generated and/or analyzed in this study are available from the corresponding author upon reasonable request.
